# Combating Malaria: Where do We Stand?

**DOI:** 10.4172/2332-0877.1000270

**Published:** 2016-02-19

**Authors:** Mamie T Coats

**Affiliations:** Department of Biological Sciences, Center for Nanobiotechnology Research, Alabama State University, AL, USA

The disease now known as malaria was described as early as 2700 B.C. In the twenty-first century, this disease remains a leading cause of morbidity and mortality. WHO statistics estimate that to date there were 214 million cases of malaria and 438000 deaths in 2015 [1]. While five *Plasmodium* species can cause malaria in humans, *Plasmodium*, *vivax* and *P. falciparum* are the predominant species in humans. *P. vivax* has the wider distribution among the two pathogens but *P. falciparum* is responsible for the majority of malaria deaths. *P. falciparum* is prevalent on the African continent. Consequently, it is here where most malaria related deaths occur.

Malaria’s ability to maintain its’ place as a major pathogen is due in part to the associated vector. The disease is transmitted by the Anopheles mosquito. The efficient predator thrives in favorable climates like that of sub-Saharan Africa. It is in this region that malaria claims most of its victims. Add to the climate the lack of adequate infrastructure to protect the most vulnerable and the result is an almost perfect storm which gives the disease the appearance of immortality.

A three-pronged approach would be the most effective way to battle malaria these include: 1) vector control/elimination, 2) rapid diagnosis and treatment of infection, and 3) prophylaxis. Each of these has met with varying degrees of success.

Vector control is accomplished through the use of insecticide-treated mosquito nets and indoor residual spraying. The usefulness of netting to prevent malaria is examined by Foumane et al. [2]. The group used surveys to examine the use and effectiveness of the National Malaria Control Program in Angola. While nets have proved effective in decreasing the activity of mosquitoes, the predominant method of mosquito control in the homes was the insecticide canister followed by mosquito coils. Households that use the nets did so largely due to the nuisance of mosquito bites and noise of the insects rather than for disease prevention. Further, homes that lacked or chose not to use nets did so because the nets are uncomfortable and not due to the cost of nets.

Indoor residual spraying with insecticides is also effective in reducing the spread of malaria. The treatment is most effective when a majority of the homes in an infected area are treated. The spraying may need to be repeated several times a year to obtain maximum effectiveness. If spraying is not supported in a majority of homes in an area the level of protection diminishes greatly.

Confirmation of malaria and rapid treatment are essentials in lowering the mortality rate of the disease. Disease should be confirmed with diagnostic testing when possible. This practice will decrease the unnecessary use of antimalarials. Along with diagnostics, attention must be given to ensure that the appropriate weight-based dosing of antimalarial therapy is used.

Current treatment recommendations for malaria include the use of artemisinin-based combination therapies (ACTs). This therapy involves the use of artemisinin and a partner drug. The significance of artemisinin was highlighted in 2015 when You you Tu received a share of the Nobel Prize in Physiology or Medicine "for her discoveries concerning a novel therapy against Malaria" [3]. Dr. Tu discovered the antimicrobial that became known as artemisinin. While ACT treatment is highly effective against susceptible organisms, resistance has been described [4,5]. Resistance to artemisinin has been described in Southeastern Asia. The resistance is identified as a delayed clearance of the parasite. Many of the mutations associated with resistance are in the propeller region of K13 of the parasite. Delayed clearance of the parasite has not been described within Africa although mutations within the propeller region of K13 are known to exist. This indicates that other factors also play a role in gaining resistance to ACTs.

In addition to traditional antimicrobials, significant attention has been given to using nanomaterials to combat malaria. Various forms of nanomaterials and nanomaterial conjugates have been examined. These materials are attractive due to their biocompatibility and versatility. A recent review by Dennis et al. examines the various nanomaterials and theirs uses with malaria [6].

Immunization against malaria has met with some recent success. A clinical trial using a recombinant form of the circumsporozoite protein of *P. falciparum* to immunize infants and children showed substantial protection over a 3–4 year period [7–9]. It is likely that investigation in negative potential side effects will happen before full-scale use of the vaccine.

Great progress in malaria treatment and prophylaxis has occurred in recent years. But there is still much room for further discovery and advancement. While the advances are having the desired effect on infection rates and mortality, surveillance programs are still needed. Accurate reports of disease must continue to share especially those with non-traditional observations. This will remind the world of the continued threat and well as maintaining the interest and research efforts of the scientific community.

Where do we stand in the fight against malaria? We stand in a world of great needs and the potential to meet the needs. The fight takes place in the arena where need and potential recognize one another and join forces. The progeny of this union eradicates malaria.
